# Analysis of new N-category on prognosis of oesophageal cancer with positive lymph nodes in a Chinese population

**DOI:** 10.2478/v10019-012-0039-6

**Published:** 2013-02-01

**Authors:** Yaping Xu, Youhua Jiang, Xinmin Yu, Qixun Chen, Xinming Zhou, Weimin Mao

**Affiliations:** 1 Department of Radiation Oncology; Zhejiang Cancer Hospital, Hangzhou, Zhejiang, China; 2 Department of Thoracic Surgery; Zhejiang Cancer Hospital, Hangzhou, Zhejiang, China; 3 Department of Medical Oncology, Zhejiang Cancer Hospital, Hangzhou, Zhejiang, China

**Keywords:** oesophageal cancer, prognostic factor, radiotherapy, oesophagectomy, chemotherapy

## Abstract

**Background:**

The 7^th^ edition of the new TNM classification system for oesophageal cancer (EC) has been published. N-category is now divided into N0, N1, N2 and N3. In this study, we aimed to validate the prognostic ability of the new N classification system in EC with positive lymph nodes in a Chinese population, and evaluate whether the new N classification system can help the decision-making for postoperative adjuvant therapy.

**Patients and methods:**

From 2002 to 2008, thoracic EC who underwent oesophagectomy were retrospectively analysed. Patients pathological stage 6^th^ edition of the American Joint Committee on Cancer / Union International Against Cancer (AJCC/UICC) TNM classification were switched to pathological stage 7^th^ edition for this analysis. Patients with pathological stage T1-4N1-3M0 EC were selected. Kaplan-Meier method and Cox regression analysis were employed to compare overall survival (OS).

**Results:**

A total of 545 patients met the inclusion criteria: 346 (63.5%) received oesophagectomy alone, 199 (36.5%) received oesophagectomy and adjuvant radiotherapy, and 36.1% (197/545) received oesophagectomy and adjuvant chemotherapy. Univariate analysis and multivariate analysis revealed significant difference in OS among patients at different postoperative pN-category (*p*<0.001). This was also present in patients receiving postoperative radiotherapy (*p*<0.001) and those undergoing postoperative chemotherapy (*p*<0.001). There was no marked difference in OS between patients receiving postoperative adjuvant therapy and surgery alone at the same postoperative pN-category, except that postoperative radiotherapy marginally improved OS in patients with pN2 and pN3 disease.

**Conclusions:**

Our results validated the prognostic ability of new N classification system. The N-category is an independent prognostic factor in patients with resectable thoracic EC who were positive for lymph nodes in a Chinese population. Further studies are required to clarify the role of new N classification system in the decision-making for postoperative adjuvant therapy.

## Introduction

Oesophageal cancer (EC) is the eighth most common cancer worldwide. In China, EC is the fourth most common cause of death and frequently found in the thorax, and 95% of EC is pathologically diagnosed as squamous cell carcinoma.^1^ Surgery, like in other thoracic malignancies^2^,^3^, is the a preferred therapeutic strategy for EC patients. But, most of patients still die of recurrence or distant metastases even in the presence of radical resection and extended lymph node dissection. Many factors have been found to affect prognosis of EC, including age, gender, tumour location, local tumour stage, degree of cell differentiation, lymph node metastasis. Among the various prognostic factors of EC, lymph node metastasis is thought to be one of the most important prognostic determinants. The overall 5-year survival rate after surgical resection is between 70% and 92% for patients without nodal involvement, but only 18–47% for patients with lymph node metastasis.^4^,^5^

The 6^th^ edition of the American Joint Committee on Cancer / Union International Against Cancer (AJCC/UICC) TNM classification for EC only defines the pathologic nodal status based on the lymph node metastasis. The 7^th^ edition of the AJCC/UICC TNM classification was published in 2010.^6^ Different from the 6^th^ edition, N-category of the 7^th^ edition were divided into pN0: no lymph node metastasis; pN1: metastasis in 1∼2 lymph nodes; pN2: metastasis in 3∼6 lymph nodes; pN3: metastasis in ≥7 lymph nodes. Increasing numbers of reports show that the numbers of positive lymph node was positively related to the prognosis.^7^–^11^

However, there are several problems to be considered before this new staging system is used to assess the prognosis of patients in Asia population. For example, most EC in Western countries is diagnosed as adenocarcinoma at the lower oesophagus or gastroesophageal junction but EC is squamous cell carcinoma in the majority of patients in Asia. Previous studies have shown that, when compared with adenocarcinoma patients, those with oesophageal squamous cell carcinoma have worse prognosis and a distinct pattern of lymphatic spread, and are susceptible to spread locally rather than systemically.^12^,^13^ In addition, lymph node dissection for EC is less performed in Western countries, whereas extended lymph node dissection is usually carried out in Asia. Thus, it is imperative to evaluate the application of this new classification system in an Asia population.

In addition, the 7^th^ edition TNM classification system for EC was revised based on the retrospective analysis of pathologic data from patients treated with primary surgical resection alone. However, patients with positive lymph nodes receiving surgery alone usually have a poor prognosis, and thus adjuvant chemotherapy and/or radiotherapy have been introduced for the therapeutic strategies. Can new N classification be used to identify patients who may require adjuvant chemotherapy and/or radiotherapy? To our best knowledge, the prognostic impact of the 7^th^ edition TNM classification system has been not evaluated in detail in EC patients undergoing postoperative adjuvant therapy.

The present study aimed to validate the prognostic ability of the new N classification system in patients with EC who were positive for lymph nodes in a Chinese population, and evaluate whether the new N classification system can help the decision-making for postoperative adjuvant therapy in this population.

## Patients and methods

### Patients

From 2002 to 2008, thoracic EC who underwent oesophagectomy were retrospectively analysed. Patients pathological stage 6^th^ edition of the American Joint Committee on Cancer / Union International Against Cancer (AJCC/UICC) TNM classification were switched to pathological stage 7^th^ edition for this analysis. Patients with pathological stage T1-4N1-3M0 EC were recruited into the present study. Other essential conditions: no distant metastasis, no preoperative chemotherapy and/or radiotherapy, no invasion to cervical oesophagus and cardiac part of the stomach.

In addition, only patients who survived for more than 3 months after surgery were included in the present study. This was done to remove possible bias in favour of the adjuvant treatment group, because some of the patients who received surgery alone may have died in the perioperative period before receiving adjuvant therapy. Thus, patients treated with oesophagectomy with or without adjuvant therapy were enrolled.

### Staging

Tumour size and extent was coded primarily according to the operative medical record and pathological findings. Number of lymph node metastasis was determined based on pathological findings. This information was used for the tumour, node, metastasis (TNM) classification according to the American Joint Committee on Cancer (AJCC) classification system (7^th^ edition). The stages of EC in these patients are shown in Table 1.

### Treatment

All patients were treated with radical resection. The standard surgical approach consisted of a limited thoracotomy on the right side and intrathoracic gastric tube reconstruction (Ivor Lewis procedure) for lesions at the middle/lower third of the oesophagus. Upper third lesions were treated by cervical anastomosis (Mckeown procedure). Most of patients underwent two-field lymphadenectomy. The number of lymph nodes harvested per case ranged from 6 to 96 (median 28). Pyloroplasty and feeding jejunostomy were not routinely done. A nasogastric tube was placed in each patient until anastomotic wound closed as assessed by oesophagography on post-operative day 14.

In this study, all patients were recommended to receive some adjuvant treatment. As the role of postoperative adjuvant radiotherapy and/or chemotherapy in the treatment of oesophageal squamous cell carcinoma was controversial at the time of treatment for these patients, the postoperative adjuvant therapy was not mandatory. The utilization of postoperative adjuvant radiotherapy and /or chemotherapy was per individual physicians’ preference and the general physical conditions. A total of 197 patients received postoperative adjuvant chemotherapy (>2 cycles). Cisplatin and 5-fluorouracil were used most frequently (67%), although several other chemotherapeutics were also used. Postoperative adjuvant radiotherapy, if given, was initiated at 4∼5 weeks after surgery. Large T-shaped field encompassing bilateral supraclavicular fossa, mediastinum and tumour bed was used. Radiation was given through anteroposterior field first to 36 Gy at 2 Gy per fraction followed by parallel opposing oblique fields to 14 Gy to avoid the spinal cord. Ten MV photons were used to deliver the radiation to the mediastinum through the anteroposterior and oblique fields. The radiation dose in all cases was prescribed to the isocenter. The bilateral supraclavicular fossas were treated with 9–12 MeV electrons. In some cases, targets were reduced on the basis of patient’s condition or physician’s judgment.

### Statistical analysis

Overall survival (OS) was determined as the time (in months) from the date of surgery to last follow-up or to September 1, 2010 for patients alive or to the date of death. Survival probability was calculated with Kaplan-Meier method and compared with log-rank test. Multivariate analysis was performed with Cox regression model. Variables in the analysis included gender, age, tumour size, pathologic T-stage, pathologic N-stage, tumour differentiation, postoperative radiotherapy and postoperative chemotherapy. Statistical analysis was performed using SPSS Version 13.0 (SPSS Inc., Chicago, IL). A value of two-sided *P*<0.05 was considered statistically significant.

## Results

### General data

A total of 545 patients were included in the present study: 346 (63.5%) received surgery alone, 199 (36.5%) postoperative radiotherapy and 197 (36.1%) postoperative chemotherapy. The average number of dissected lymph nodes was 31.2±17.4 (mean±SD) nodes per case (median 28, range 6–96). The mean number of metastatic nodes was 4.1±1.9 (median 2, range 1–36). According to the 7^th^ edition of TNM classification system, pN1 EC was found in 270 cases, pN2 EC in 188 and pN3 EC in 87 cases. The median age was 57 years (range: 36∼86 years). Median follow-up period for survived patients was 51 months (range 4∼93 months). The patients’ characteristics are presented in Table 2. Males, and those aged <65 years, or with tumor length >5 cm or having more positive lymph nodes are more likely to receive postoperative adjuvant therapy.

### Overall survival

The median survival time was 26.5 months, and the 3-year OS rate was 41.0%. The postoperative radiotherapy was found to be significantly associated with improved OS (*p* = 0.006). The median survival time was 31 months in patients receiving postoperative radiotherapy and 21 months in those undergoing surgery alone. The postoperative radiotherapy dramatically improved OS at 3 years from 38.3 to 45.8% when compared with surgery alone. However, the median survival time was decreased from 28 months to 23 months in patients receiving postoperative chemotherapy as accompanied by a decrease in 3-year OS from 43.1 to 37.1%, but without significant differences (*p* = 0.508).

### Overall survival by (7^th^ edition) AJCC N classification grouping

**FIGURE 1.**
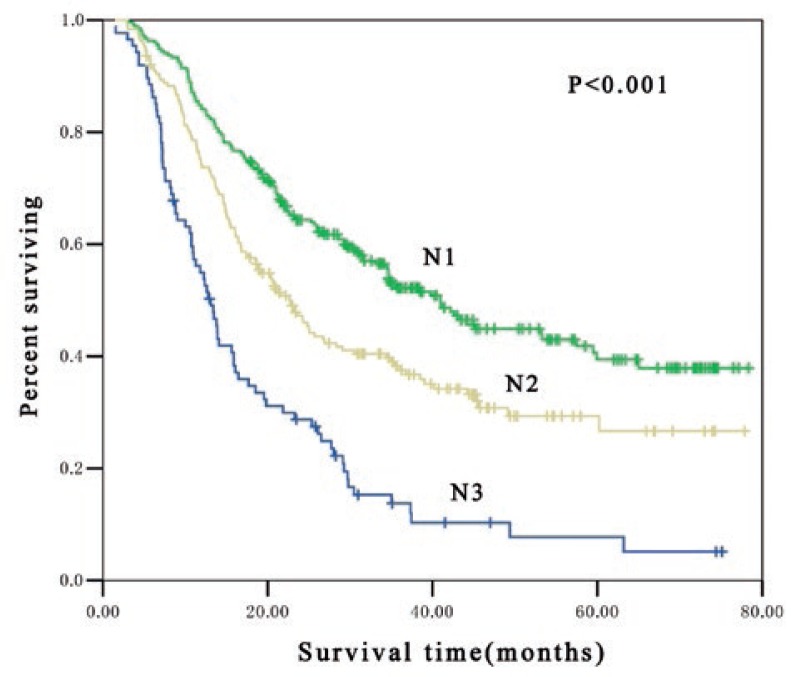
Kaplan-Meier estimates for overall survival of 545 patients stratified by N classification. The median survival time was 41 months, 23 months and 13 months for pN1, pN2 and pN3, respectively (*p* < 0.001).

**FIGURE 2.**
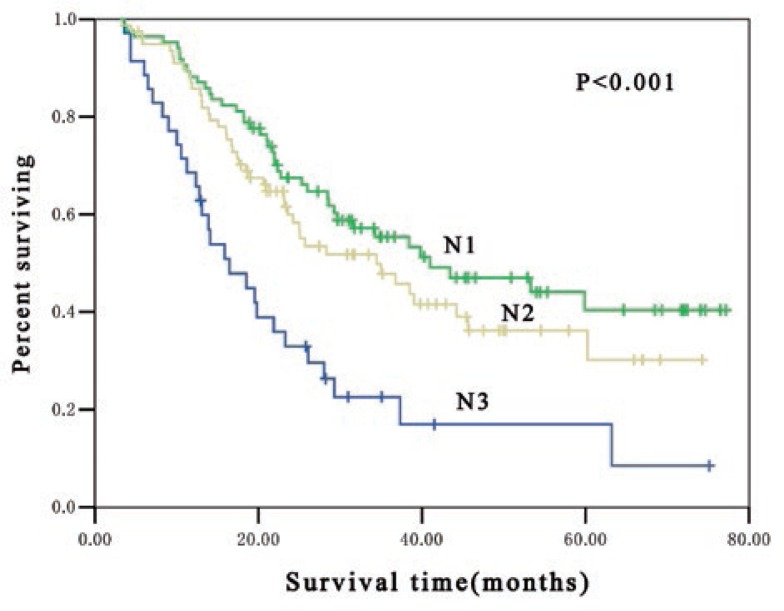
Kaplan-Meier estimates for overall survival of 199 postoperative adjuvant radiotherapy patients stratified by N classification. The median survival time was 41 months, 34 months and 16 months for pN1, pN2 and pN3, respectively (*p* < 0.001).

**FIGURE 3.**
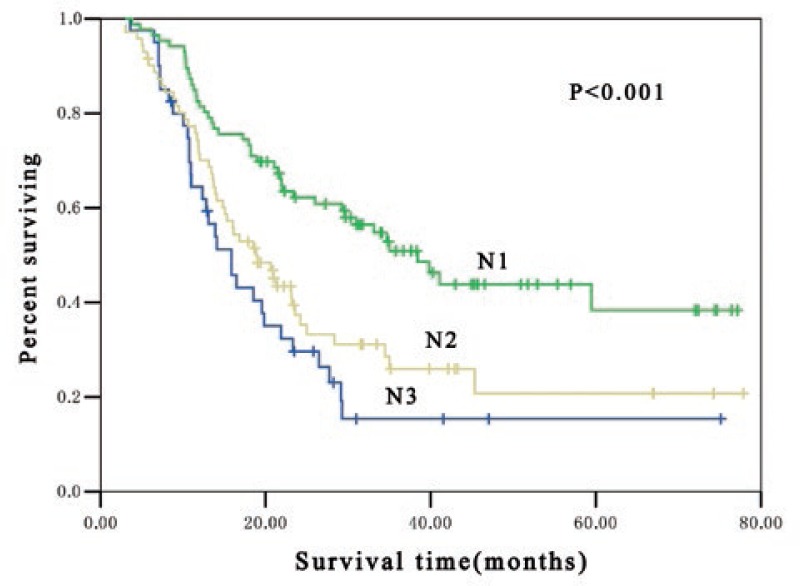
Kaplan-Meier estimates for overall survival of 197 postoperative adjuvant chemotherapy patients stratified by N classification. The median survival time was 38 months, 19 months and 16 months for pN1, pN2 and pN3, respectively (*p* < 0.001).

In pathologic lymph nodes positive patients (pN1, pN2 and pN3), significant difference in OS were found among patients at different postoperative pN-category. The median survival time for pN1, pN2 and pN3 patients were 41 months, 23 months and 13 months, respectively (*p* < 0.001) and the 3-year OS rate was 51.5%, 37.5% and 13.8%, respectively (*p* < 0.001) (Figure 1). Subgroup analysis indicated that there was significant difference in OS among patients at different postoperative pN-category who received postoperative adjuvant radiotherapy. In these patients, the median survival time for pN1, pN2 and pN3 patients were 41 months, 34 months and 16 months, respectively (*p* < 0.001) and the 3-year OS rate was 53.3%, 45.7% and 16.9%, respectively (*p* < 0.001) (Figure 2). Furthermore, marked difference was also noted in OS among patients at different postoperative pN-category who underwent postoperative adjuvant chemotherapy. The median survival time for pN1, pN2 and pN3 patients were 38 months, 19 months and 16 months, respectively (*p* < 0.001) and the 3-year OS rate was 50.9%, 31.2% and 15.4%, respectively (*p* < 0.001) (Figure 3).

There was no marked difference in OS between patients receiving postoperative radiotherapy and surgery alone at the same postoperative pN-category. For patients undergoing surgery alone and those receiving concomitant postoperative radiotherapy, the 3-year OS was 50.8% and 53.3%, respectively, in patients at pN1 (*p* = 0.507); 31.4% and 45.7%, respectively, in those at pN2 (*p* = 0.080); 8.6% and 16.9%, respectively, in those at pN3 (*p* = 0.064), only marginally improved OS for pN2 and pN3 disease. This was also noted between patients receiving postoperative chemotherapy and surgery alone. For patients receiving surgery alone and those undergoing concomitant postoperative chemotherapy, the 3-year OS was 52.8% and 50.9%, respectively, in patients at pN1 (*p* = 0.768); 40.6% and 31.2%, respectively, in those at pN2 (*p* = 0.570); 10.2% and 15.4%, respectively, in those at pN3 (*p* = 0.131) (Table 3).

### Univariate and multivariate analyses

On unvariate analysis, the number of positive lymph nodes (pN2/pN1: hazard ratio [HR] 1.56, 95% confidence interval [CI] 1.22–1.99, *p* < 0.001; pN3/pN1: HR 3.19, 95% CI 2.40–4.23, *p* < 0.001) was associated with survival. Postoperative radiotherapy was associated with improved survival (HR 0.79, 95% CI 0.63–0.99, *p* = 0.045). Male gender and high T stage predicted a decrease of OS (Table 4).

On multivariate analysis, the number of positive lymph nodes was also associated with survival (pN1/pN3: HR 1.55, 95% CI 1.20–2.00, *p* = 0.001; pN2/pN3: HR 2.27, 95% CI 1.45–3.54, *p* < 0.001). Postoperative radiotherapy was associated with improved survival (HR 0.71, 95% CI 0.55–0.87, *p* = 0.001). Male gender and high T stage predicted a decrease of OS (Table 5).

## Discussion

The oesophageal cancer TNM classification system was recently revised in the 7^th^ edition of AJCC/UICC) staging system. The major differences between the 6^th^ and 7^th^ editions include: (1) T is re-defined and T4 subclassified as T4A and T4B; (2) the regional lymph nodes are re-defined. N is subclassified according to the number of positive regional lymph nodes; (3) M is re-defined. In addition, prognostic staging, including histological grade and cancer location, are defined for T1-3N0M0 patients. However, the reliability and accuracy of the nodal portion of the TNM staging system still remains the much debate. Several studies have evaluated the influence of the number of lymph node metastasis on the prognosis of patients with esophageal cancer and have found significant differences in prognosis between the patients with different numbers.^7^–^10^ However, several authors have criticized the new N classification, claiming that it omits some potentially critical prognostic information relating to lymph node status.^14^ The present study showed that, according to the 7^th^ edition TNM classification system, N classification independently affected the prognosis of thoracic EC patients with positive lymph nodes after radical surgery in Chinese population. This was also present in patients receiving postoperative radiotherapy (*p* < 0.001) and those undergoing postoperative chemotherapy (*p* < 0.001). Lymph node involvement is a significant prognostic indicator of overall survival, and as the number of involved nodes increases, it may suggest a greater tumour burden or more aggressive tumour biology, and so the likelihood of locoregional or systemic recurrence could be assumed to be higher.

Cancer staging systems aim to predict survival and then provide information for the selection of therapeutic strategies. The high incidence of regional recurrence of EC suggests that surgery alone is not effective enough in such cases and adjuvant therapy is indicative. However, up to now, whether postoperative radiotherapy and chemotherapy affect the therapeutic outcomes remains controversial.^15^–^20^ Therefore, in those for whom the primary treatment is surgery, there is no clear indication for adjuvant therapy. It is necessary to generate criteria to help the decision-making for postoperative adjuvant therapy in this population. In the 7^th^ edition, the postoperative pN-categories are re-defined based on the number of positive regional lymph nodes. Many studies have addressed the impact of postoperative radiotherapy on the prognosis of oesophageal squamous cell carcinoma patients based on the number of positive lymph nodes. Xiao *et al*.^17^ randomized 495 patients with oesophageal squamous cell carcinoma to radical resection alone vs. postoperative radiotherapy (a total of 50∼60 Gy). There was no survival benefit with addition of postoperative radiotherapy (a 5-year OS of 31.7% for surgery alone *vs.* 41.3% for postoperative radiotherapy; *p* = 0.4474). However, when patients were stratified based on the number of positive lymph nodes, obvious survival benefit was noted in patients receiving postoperative radiotherapy with an improvement in 5-year OS from 17.6 to 34.1%, (*p* = 0.0378). In patients with 1∼2 positive lymph nodes, the 5-year survival rate was 23.5% in the surgery alone group and 45.1% in the postoperative radiotherapy group (*p* = 0.1286). In patients with ≥3 positive lymph nodes, the 5-year survival rate was 0% and 20.6% in the surgery alone group and postoperative radiotherapy group, respectively (*p* = 0.0265). Chen *et al*.^21^ retrospectively evaluated patients with thoracic oesophageal squamous cell carcinoma, their results showed the postoperative radiotherapy was significantly associated with improvement in survival, which was only observed in patients with ≥3 positive lymph nodes. Similar to the findings above mentioned, our study revealed that postoperative radiotherapy marginally improved OS of patients with thoracic EC at pN2 and pN3 disease. Adjuvant radiotherapy can theoretically treat microscopic disease left behind after an incomplete surgery and increase local control. Therefore, we postulate that the new N classification system may potentially help the decision-making for postoperative adjuvant radiotherapy. Given the insufficient evidence, adequately powered prospective randomized trials are required to confirm these findings. Thus, caution should be taken before applying these findings in clinical practice.

Ando *et al.*^20^ conducted a randomized multi-center trial to determine whether postoperative adjuvant chemotherapy improves the outcome of patients with esophageal squamous cell carcinoma who underwent radical surgery. Their results showed that postoperative adjuvant chemotherapy with cisplatine and fluorouracil significantly prevented relapse of EC when compared with surgery alone. The theoretical advantages of adding adjuvant chemotherapy to the treatment of esophageal cancer are for potential targeting micrometastatic disease, thus decreasing the risk of distant spread. Nevertheless, our study showed that the therapeutic efficacy of adjuvant chemotherapy was unsatisfactory, even for patients with pN3 patients. This might be attributed to the fact that our study was retrospective and the chemotherapy regimens were not quite the same among these patients. Therefore, the therapeutic efficacy of postoperative adjuvant chemotherapy in these patients is still no clear indication regardless of EC at pN1, pN2 or pN3 based on our data. More studies are required to confirm the role of adjuvant chemotherapy.

The weakness of this study can be summarized as follows: First, only patients with squamous cell carcinoma were recruited in this study. In contrast, the incidence of adenocarcinoma is dramatically increasing in Western countries. Therefore, the results of this study may be not generalized to be applied in North American and European patients. In addition, on univariate and multivariate analysis, male gender and increased number of positive lymph nodes were significantly associated with worse outcome (Tables 4, 5). However, males and those having more positive lymph nodes were more likely to receive postoperative radiotherapy and chemotherapy (Table 2), likely providing a further bias against improved survival in receiving postoperative adjuvant therapy patients. Moreover, in a phase II non-randomized trial which evaluated postoperative concurrent chemoradiation with cisplatin and 5-fluorouracil in patients with poor prognosis oesophageal and gastroesophageal junction (EGJ) cancers, the projected rates of 4-year overall survival, freedom from recurrence, distant metastatic control and locoregional control were 51%, 50%, 56% and 86% respectively for patients with lymph node positive (T3 or T4) tumours, which are better than the historical outcomes with surgery alone.^22^ However, the efficacy of postoperative chemoradiation has not been compared to surgery alone in a randomized trial in patients with EC. Therefore, evaluation of postoperative chemoradiotherapy for these patients may be important. Further development of postoperative adjuvant therapy in EC is warranted.

In summary, the results of our study demonstrate that the new N-category in the 7^th^ edition of AJCC/UICC TNM classification system are an independent prognostic factor in lymph node positive patients with thoracic EC in a Chinese population. However, the evidence is not powerful enough to support that the new N classification system can help the decision-making for postoperative adjuvant therapeutic strategy in these patients.

## Figures and Tables

**TABLE 1.** Patient characteristics based on TNM classification and AJCC stage grouping

	**Stage grouping**	**PORT**	**POCT**	**Surgery alone**
T1-2N1	II B	13	16	41
T1-2N2	IIIA	16	12	11
T3N1	IIIA	70	68	132
T3N2	IIIB	54	51	91
T4N1-3	IIIC	46	50	71
Total No. of patients		199	197	346

TNM = tumour, node, metastases based classification; AJCC = American Joint Committee on Cancer; PORT = postoperative radiation therapy; POCT = postoperative chemotherapy

**TABLE 2.** Comparison of patient characteristics by treatment assignment (N=545)

**Variable**	**All patients (%)**	**PORT**			**POCT**		

		**Yes**	**No**	**p^a^**	**Yes**	**No**	**p^a^**
Gender				0.024			0.012
Female	55(10)	13(7)	42(12)		12(6)	43(12)	
Male	490(90)	186(93)	304(88)		185(94)	305(88)	
Age				0.018			<0.001
<65	421(77)	164(82)	257(74)		175(89)	246(71)	
≥65	124(23)	35(18)	89(26)		22(11)	102(29)	
Tumour length				0.030			0.020
<5cm	274(50)	89(45)	185(53)		87(44)	187(54)	
≥5cm	271(50)	110(55)	161(47)		110(56)	161(46)	
T-stage				0.784			0.764
T1–2	93(17)	33(16)	60(17)		34(17)	59(17)	
T3	407(75)	149(75)	258(75)		149(76)	258(74)	
T4	45(8)	17(9)	28(8)		14(7)	31(9)	
N-stage				0.042			0.015
N1	270(50)	85(13)	185(53)		86(44)	184(53)	
N2	188(34)	79(40)	109(32)		71(36)	117(34)	
N3	87(16)	35(17)	52(15)		40(20)	47(13)	
Tumour differentiation				0.428			0.342
High (G1)	78(14)	33(16)	45(13)		24(12)	54(16)	
Moderate (G2)	347(64)	123(62)	224(65)		124(63)	223(64)	
Low (G3)	120(22)	43(22)	77(22)		49(25)	71(20)	

a = *X*^2^ *p*-value; PORT = postoperative radiation therapy; POCT = postoperative chemotherapy.

**TABLE 3.** Overall survival based on 7th AJCC N-stage grouping

**Variable**	**All patients (%)**	**PORT**				**POCT**			

		**Yes/No**	**No. (%)**	**3-yr OS (%)**	**p**	**Yes/No**	**No. (%)**	**3-yr OS (%)**	**p**
N1	270(50)	Yes	85(43)	53.3	0.507	Yes	86(44)	50.9	0.768
		No	185(53)	50.8		No	184(53)	52.8	
N2	188(34)	Yes	79(40)	45.7	0.080	Yes	71((36)	31.2	0.570
		No	109(32)	31.4		No	117(34)	40.6	
N3	87(16)	Yes	35(17)	16.9	0.064	Yes	40(20)	15.4	0.131
		No	52(15)	8.6		No	47(13)	10.2	

PORT = postoperative radiotherapy; POCT = postoperative chemotherapy; OS = overall survival

**TABLE 4.** Univariate analysis for survival

**Variable**	**CHR**	**95% CI**	**p**
Gender			
Female	1		
Male	2.04	1.31–3.17	0.002
Age			
<65	1		
≥65	1.16	0.90–1.48	0.254
Tumor length			
<5cm	1		
>5cm	1.06	0.85–1.31	0.600
T-stage			
T1–2	1		
T3	1.37	1.00–1.86	0.049
T4	2.60	1.69–4.01	<0.001
N-stage			
N1	1		
N2	1.56	1.22–1.99	<0.001
N3	3.19	2.40–4.23	<0.001
Tumor differentiation			
High (G1)	1		
Moderate (G2)	0.87	0.63–1.19	0.369
Low (G3)	1.19	0.83–1.70	0.339
PORT			
Yes	0.79	0.63–0.99	0.045
No	1		
POCT			
Yes	1.08	0.86–1.35	0.509
No	1		

CHR = Cox hazard ratio; 95% CI = 95% Confidence Interval; PORT = postoperative radiation therapy; POCT = postoperative chemotherapy

**TABLE 5.** Mutivariate analysis for survival

**Variable**	**CHR**	**95% CI**	**p**
Gender			
Female	0.56	0.36–0.88	0.012
Male	1		
Age			
<65	1		
≥65	1.04	0.80–1.34	0.783
Tumor length			
<5cm	1		
≥5cm	0.97	0.78–1.21	0.785
T-stage			
T1–2	1		
T3	1.35	0.99–1.85	0.059
T4	2.15	1.37–3.36	0.001
N-stage			
N1	1		
N2	1.55	1.20–2.00	0.001
N3	2.27	1.45–3.54	<0.001
Tumor differentiation			
High (G1)	1		
Moderate (G2)	0.85	0.62–1.17	0.319
Low (G3)	1.00	0.70–1.44	0.993
PORT			
Yes	0.71	0.55–0.87	0.001
No	1		
POCT			
Yes	1.20	0.92–1.55	0.163
No	1		

CHR = Cox hazard ratio; 95% CI = 95% Confidence interval; PORT = postoperative radiation therapy; POCT = postoperative chemotherapy
